# The Regional Student Group Program of the ISCB Student Council: Stories from the Road

**DOI:** 10.1371/journal.pcbi.1003241

**Published:** 2013-09-26

**Authors:** Geoff Macintyre, Magali Michaut, Thomas Abeel

**Affiliations:** 1 NICTA Victoria Research Laboratory, University of Melbourne, Parkville, Victoria, Australia; 2 Bioinformatics and Statistics, the Netherlands Cancer Institute, Amsterdam, The Netherlands; 3 Genome Sequencing and Analysis Program, Broad Institute of MIT and Harvard, Cambridge, Massachusetts, United States of America; 4 VIB Department of Plant Systems Biology, UGent, Ghent, Belgium

## Abstract

The International Society for Computational Biology (ISCB) Student Council was launched in 2004 to facilitate interaction between young scientists in the fields of bioinformatics and computational biology. Since then, the Student Council has successfully run events and programs to promote the development of the next generation of computational biologists. However, in its early years, the Student Council faced a major challenge, in that students from different geographical regions had different needs; no single activity or event could address the needs of all students. To overcome this challenge, the Student Council created the Regional Student Group (RSG) program. The program consists of locally organised and run student groups that address the specific needs of students in their region. These groups usually encompass a given country, and, via affiliation with the international Student Council, are provided with financial support, organisational support, and the ability to share information with other RSGs. In the last five years, RSGs have been created all over the world and organised activities that have helped develop dynamic bioinformatics student communities. In this article series, we present common themes emerging from RSG initiatives, explain their goals, and highlight the challenges and rewards through specific examples. This article, the first in the series, introduces the Student Council and provides a high-level overview of RSG activities. Our hope is that the article series will be a valuable source of information and inspiration for initiating similar activities in other regions and scientific communities.

## The RSG Article Series

This article series draws on the collective experience of the ISCB Student Council to demonstrate the effectiveness of a Regional Student Group (RSG) program. The goal of the series is to inspire students in the broader community to start similar initiatives. Each of the articles is written by authors from various RSGs that have had hands-on experience in the article topic. The articles share both recipes that worked well and formulas that did not. The topics covered include: interactive science, scientific meetings, career development, and the challenges and benefits of running and starting a Regional Student Group. See Table 1 for an outline of the series.

**Table 1:** This table provides an overview of the articles that are part of the article series on the ISCB Student Council Regional Student Group program.

Topic	Article Title
Interactive science	Explain bioinformatics to your grandmother!
	Breaking the ice and forging links: The importance of socialising in science
	The spirit of competition: To win or not to win
Scientific meetings	Building the future of bioinformatics through student facilitated conferencing
	Workshops: A great way to enhance and supplement a degree
Career development	Paving the way towards a successful and fulfilling career
	Learn from the best
Running an RSG: The challenges and benefits	Crossing borders for science
	Soft skills: An important asset acquired from RSG organized activities
	Failure, that troublesome obstacle in the way to success: The RSG files
	Establishing and managing a global student network

## Introducing the ISCB Student Council

The International Society for Computational Biology (ISCB) is a professional society whose aim is to help in the advancement of scientific understanding of living systems through computation. Founded in 1997, today there are over 3,000 members across more than 50 countries. The activities of the ISCB over the years have contributed to creating a critical mass of researchers in the field of computational biology.

As part of the ISCB, the Student Council is a student society operated by student volunteers. The Student Council was created in 2004 when a group of students discussed how they could better help the community of young researchers in the field of computational biology. Following a small symposium to gauge interest, the ISCB Student Council was formed as a student organization lead by and for students. The Student Council's goal is to promote the development of the next generation of computational biologists. Over the years, the council has grown and worked on its mission through provision of scientific events, networking opportunities, soft-skills training, educational resources, and career advice, while attempting to influence policy processes affecting science and education.

After bringing together young scientists from many countries through some of the early ISCB Student Council Symposia [1]–[6], the council gathered valuable feedback from its members about the needs and expectations of a student society in computational biology. It became apparent that students in different parts of the world had vastly different needs. For example, students in Africa were requesting basic bioinformatics training workshops due to a lack of formal education options at the institutes in the region, whereas European students were seeking a way to connect with their peers given the scarcity of researchers in the area at the time. Because there was a critical mass of interested students in each region, it was agreed that a program was needed that allowed students to form regional affiliates, which addressed the specific needs of students in that region. Thus, the ISCB Student Council Regional Student Group program was born.

## The Regional Student Group Program

**Figure 1:**
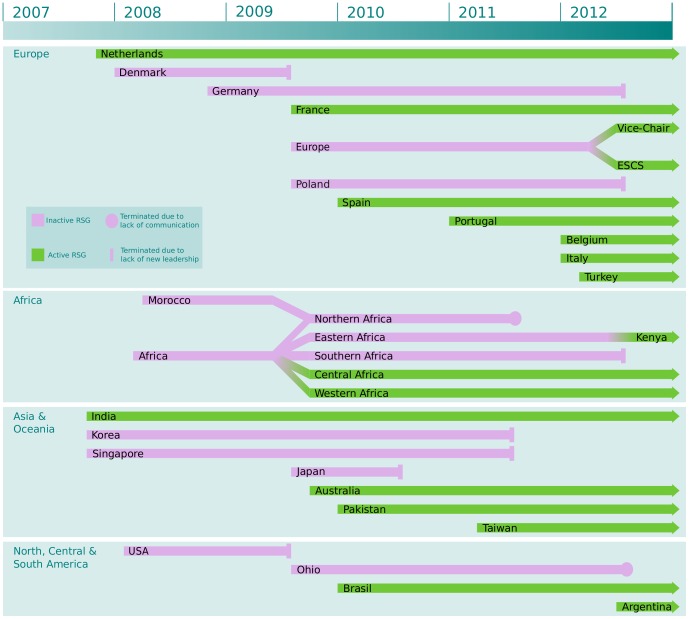
This figure depicts the evolution of Regional Student Groups as part of the RSG program of the International Society for Computational Biology Student Council. RSGs are grouped by geographical region. Those highlighted in green are active RSGs, while those in purple are no longer active. Information to get started with your own RSG, or to restart one of the dormant ones, is available from the SC website (http://www.iscbsc.org/content/regional-student-groups).

Since the RSG program was started in 2006, over 25 new RSGs have been created (see Figure 1 for an overview). Each new RSG has had a measurable impact on the student communities in each region, and the RSG activities and initiatives have benefited members as well as organisers. Creating a new Student Council RSG has proved relatively easy. It usually only requires a small number of enthusiastic students to form a diverse team that is representative of the geographic region. The team ideally comprises students from different universities in the area. In addition, a faculty advisor is required. The role of the faculty advisor is to provide expert advice and access to resources and a large network of established scientists. Once the team is formed, it is then a matter of running two or more activities to mobilise the group and build membership. A large event followed by a smaller event provides a good balance between the amount of time invested by volunteers and the usefulness in building a community. After following these points, an RSG is generally up and running in a few months and gaining more members each day.

## RSG Activities

RSGs have organised many events and initiated important activities since the inception of the RSG program. These range from traditional events such as a symposium [1]–[6], to relaxed and informal social events, such as scientific speed-dating [5]. RSG-organised symposia or conferences promote interactions between students and give them the opportunity to present and discuss their work. As these events are typically smaller than an international conference, the environment can be less intimidating. It also gives the organisers insight into how to operate a large-scale conference.

Another popular RSG activity is a student-run workshop. These provide members with opportunities to learn new techniques through hands-on training. Depending on the local needs of the student group, these workshops may be technical-skill driven, such as programming in Python, or soft-skill oriented, such as the art of presenting scientific work. During the course of these events, there is ample opportunity to practice and interact with the workshop tutors.

In addition to these typical professional academic activities, several RSGs have organized more creative events: a writing competition for articles in popular science magazines, exercises in popularizing science for lay people, or breakfast with coffee, French pastries, and an expert.

It is the consensus amongst members however, that the most important career-building part of RSG activities are the social interactions. These have taken the form of breakfast meetings, scientific speed dating sessions, or simply informal discussions over drinks. Basically any event that creates an environment in which students can feel comfortable connecting with others results in a successful event. Quite often, the best way to connect scientists is to encourage them to discuss something completely unrelated to their science. The importance of a good connection cannot be underestimated, and throughout the series this will be a recurring element. Building a network through RSG events provides links with future collaborators, something that can truly further a career.

Each and every one of the activities outlined above, and in this article series, has been organised by a local team of engaged people as part of an RSG. In many cases, the event was organised because there was no one else offering something similar. Because event organisers are working to fulfil the needs of their local community, they often do an excellent job with minimal resources and gain valuable skills along the way. However, sometimes things do not pan out as planned, especially for those RSGs attempting to run a novel style of event. One of the strengths of the program is, however, that RSGs can learn from their mistakes and share their knowledge. Once a certain RSG has worked out the correct “formula” for running an event, they can share this approach with other RSGs. This works particularly well for events that operate in all regions, such as symposia and social events.

The articles in this series provide detailed descriptions of these activities, tips on how to run them, and the associated challenges and benefits.

## The Challenges and Rewards of Participating in an RSG

RSG operations are designed to supplement and enhance formal education in bioinformatics and computational biology at a local level. The activities organised by RSGs provide students attending them with a range of experience in “soft skills” such as communication and presentation. This results in a well-rounded tertiary education experience, which provides students with a competitive advantage in their future careers. Being involved in an RSG as an organiser provides students with collaboration, organisation, and coordination skills. This involvement may take substantial time and effort, but it brings substantial benefits [7]. Students involved in the RSG organisation committee start by building a network within the core of the RSG team. Working together and organising several events creates strong links that typically lead to scientific collaboration and/or friendship. The network students build is also not restricted to the RSG team, but can extend to the broader national and international communities via the link with the Student Council and other RSGs. The series articles under the topic “Running an RSG: the challenges and benefits” (Table 1) provide more detail in this area.

## Running the Regional Student Group Program

During the lifespan of the ISCB Student Council RSG program, a number of RSGs have gone dormant (See Figure 1). In some cases, RSGs were simply merged or split, but in other cases the RSG went dormant because there was a lack of new student volunteers available to take over core RSG committee positions. While unfortunate, these experiences have provided an opportunity to refine the RSG program to ensure successful and continued operation of all new RSGs in the future.

During the operation of the program, the Student Council has identified a number of issues that have made things difficult for volunteers and has developed strategies to avoid them. For more details see the articles as part of “Running an RSG: the challenges and benefits” (Table 1).

In brief, a key issue causing problems in the program is a lack of critical mass of motivated students in the local community. This results in a lack of volunteers to run new events or to take over when leaders retire. To help overcome this issue, the Student Council has developed a peer-reviewed application and renewal process for RSGs. Each year the progress of an RSG can be tracked and advice can be given on how to maintain a critical mass of motivated students through employing the correct activities and initiatives.

Another key issue is RSGs over-planning without delivering. A group of motivated students can come up with a large number of ideas for events to run, but it is much harder to anticipate what is involved in putting these events into practice. This is why the Student Council encourages new RSGs to initially run a small number of events to gauge how much work is involved. It is then easier for the RSG to expand as repeat events generally involve much less effort. This results in RSGs delivering their proposed events after a small amount of experience.

The final key issue is the potential for an RSG to lack geographic or institutional diversity in the team. While it is convenient to have everybody running an RSG in the same city, or institute, it makes it difficult for students from other places to get involved. This can easily result in a lack of critical mass of motivated students, which takes us back to the first issue that was raised. Therefore, as part of the renewal process, all RSGs must show institution diversity in the core committee.

With strategies in place to avoid these issues, and with the Student Council on the lookout for any others, we anticipate that the RSG program will continue to run successfully in the future.

## Conclusion

The articles in this series provide details on all of the activities and issues raised in this introductory piece. It is the ISCB Student Council's hope that this series will motivate others to start similar initiatives so that more students can experience the amazing benefits that come with being part of a Regional Student Group program.

**About the Authors**

The authors have worked closely together for many years on many aspects of the ISCB Student Council and the Regional Student Group program.

**Geoff Macintyre** was co-founder and president (2009–2010) of RSG-Australia and served as ISCB Student Council chair from January 2011 to January 2013.

**Magali Michaut** was co-founder and president (2008–2010) of RSG-France and served on their Board of Directors from 2008 to 2013. She was also co-founder, secretary (2009), and president (2010–2011) of RSG-Europe. She served as secretary for the ISCB Student Council in 2009.

**Thomas Abeel **was co-founder, president (2009) and secretary (2010–2011) of RSG-Europe and served as Student Representative to the ISCB Board of Directors from January 2011 to January 2013.
